# Deletion of Galectin-3 Enhances Xenobiotic Induced Murine Primary Biliary Cholangitis by Facilitating Apoptosis of BECs and Release of Autoantigens

**DOI:** 10.1038/srep23348

**Published:** 2016-03-21

**Authors:** Aleksandar Arsenijevic, Marija Milovanovic, Jelena Milovanovic, Bojana Stojanovic, Natasa Zdravkovic, Patrick S.C. Leung, Fu-Tong Liu, M. Eric Gershwin, Miodrag L. Lukic

**Affiliations:** 1 Center for Molecular Medicine and Stem Cell Research, Faculty of Medical Sciences, University of Kragujevac, Serbia; 2 Department of Internal Medicine, Faculty of Medical Sciences, University of Kragujevac, Serbia; 3 Division of Rheumatology, Allergy and Clinical Immunology, University of California at Davis School of Medicine, Davis, CA, USA

## Abstract

Galectin-3 (Gal-3) is a carbohydrate binding lectin, with multiple roles in inflammatory diseases and autoimmunity including its antiapoptotic effect on epithelial cells. In particular, increased expression of Gal-3 in epithelial cells is protective from apoptosis. Based on the thesis that apoptosis of biliary epithelial cells (BECs) is critical to the pathogenesis of Primary Biliary Cholangitis (PBC), we have analyzed the role of Gal-3 in the murine model of autoimmune cholangitis. We took advantage of Gal-3 knockout mice and immunized them with a mimotope of the major mitochondrial autoantigen of PBC, 2-octynoic acid (2-OA) coupled to BSA (2OA-BSA) and evaluated the natural history of subsequent disease, compared to control wild-type mice, by measuring levels of antibodies to PDC-E2, immunohistology of liver, and expression of Gal-3. We report herein that deletion of Gal-3 significantly exacerbates autoimmune cholangitis in these mice. This is manifested by increased periportal infiltrations, bile duct damage, granulomas and fibrosis. Interestingly, the BECs of Gal-3 knockout mice had a higher response to apoptotic stimuli and there were more pro-inflammatory lymphocytes and dendritic cells (DCs) in the livers of Gal-3 knockout mice. In conclusion, Gal-3 plays a protective role in the pathways that lead to the inflammatory destruction of biliary epithelial cells.

Gal-3, a member of the β-galactoside-binding lectin family, is expressed in various tissues and cell types. Gal-3 modulates numerous cellular functions and can be found in the cytoplasm, the nucleus, on the surface of cells and in extracellular space^1^. ^1^Extracellular Gal-3 modulates cell adhesion to different extracellular matrix components by lattice formation and cross-linking of matrix molecules and cell surface glycoproteins. Furthermore, extracellular Gal-3 can modulate signaling pathways by binding to cell surface ligands and promotes apoptosis. On the other hand, intracellular Gal-3 suppresses apoptosis, promotes cell growth, and can regulate signal transduction. In the nuclei, Gal-3 exhibits transcriptional activity and promotes proliferation of cells^2^. Intranuclear Gal-3 overexpression can be found in many types of cancers. Gal-3 is also involved in the pathogenesis of many chronic inflammatory and malignant diseases^3^,^4^,^5^,^6^,^7^,^8^. Epithelial cells of normal intrahepatic bile ducts constitutively, but weakly express Gal-3, while its expression is strongly increased in intrahepatic cholangiocarcinoma^9^,^10^. Intracellular Gal-3 in epithelial cells have anti-apoptotic effects^9^. For example, increased expression of Gal-3 in keratinocytes after their exposure to UV light protected them from apoptosis^11^.

These observations on Gal-3 are particularly noteworthy for PBC, which is characterized by a multi-lineage response to the major mitochondrial autoantigen, PDC-E2. There have been extensive studies on the natural history of PBC, including the multiple pathways that lead to immunopathology in both humans and mice^12^,^13^,^14^,^15^,^16^,^17^,^18^,^19^,^20^,^21^,^22^,^23^,^24^,^25^. These data illustrate several principles. Firstly, genetic predisposition plays a critical role. Secondly, both innate and adaptive responses are involved at different stages of disease. Thirdly, women are more commonly affected. Though this finding remains unexplained, it is suggested to be a consequence of both hormonal and genetic factors including epigenetic events on the X chromosome^26^,^27^,^28^,^29^,^30^,^31^,^32^.

An interesting thesis on PBC is that BECs are not passive bystanders in PBC. Through variable expression of adhesion molecules, costimulatory molecules and proinflammatory cytokines, BECs can modulate the intensity of the inflammatory processes upon stimulation^33^. BECs are also susceptible to apoptosis^34^. During this process the major mitochondrial autoantigen, PDC-E2, remains immunologically intact^35^ and is expressed at the luminal surface of the small bile duct cells^36^. Autoreactive lymphocytes can be activated by antigen(s) originating from apoptosomes released from BECs^37^. Apoptotic BECs can also stimulate the release of proinflammatory cytokine from monocyte-derived macrophages^38^.

Animal models of PBC include the immunization of C57BL/6 mice with 2-octynoic acid (2-OA) coupled to BSA, which is characterized by high titer of anti mitochondrial antibodies (AMAs), portal inflammation, and immune mediated cholangitis similar to human PBC^39^. We report herein that in this experimental model of PBC, Gal-3 deletion exacerbates the natural history of disease, including portal inflammation and fibrosis. We submit that this is the result of enhanced release of autoantigen and an increase in stimulation of antigen presenting cells.

## Results

### BECs expression and serum level of Gal-3 is increased in PBC patients

**Figure 1:**
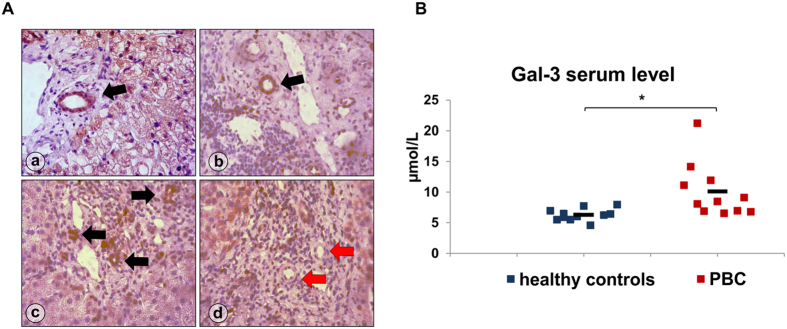
Gal-3 is overexpressed in BECs and level of Gal-3 is increased in the serum PBC patients. (**A**) Immunohistochemistry of Gal-3 in liver pathology a) PBC, b) viral hepatitis B, c), viral hepatitis C, d) sclerosing cholangitis (Gal-3 expression in BECs, black arrows; no Gal-3 expression in sclerosing bile ducts, red arrows). (**B**) Serum level of Gal-3 determined in eleven patients irrespectively of histological stage of disease and eleven healthy age and sex matched controls. Data were analyzed by Student t test and presented as mean + SE, or sccater plot *p < 0.05.

We have previously shown that Gal-3 is expressed very weakly in the biliary epithelial cells and liver parenchyma in healthy controls but is strongly expressed in patients with drug and virus induced hepatitis (7). To understand the role of Gal-3 in human PBC, we examined the expression of Gal-3 in liver tissue sections of patients diagnosed with PBC. Gal-3 overexpression was observed in the cytoplasm and nucleus of BECs in patients with PBC (Fig. 1A). Additionally, serum level of Gal-3 was significantly higher in the group of eleven PBC patients compared to eleven paired healthy controls (p < 0.05) (Fig. 1B). As in previous studies there was no significant expression of Gal-3 in healthy livers (7). However, high levels of Gal-3 expression were also detected in BECs of patients with viral hepatitis B and C. Furthermore, intracytoplasmic and intranuclear overexpression of Gal-3 in BECs of patients with viral hepatitis B and C was also accompanied by intra-hepatocyte and extracellular overexpression of Gal-3 around inflammatory infiltrates. On the other hand, Gal-3 was mostly absent in BECs of patients with sclerosing cholangitis, was and is only weakly expressed in the cytoplasm.

### Gal-3 deficiency exacerbates primary biliary cholangitis induced with 2OA-BSA

**Figure 2:**
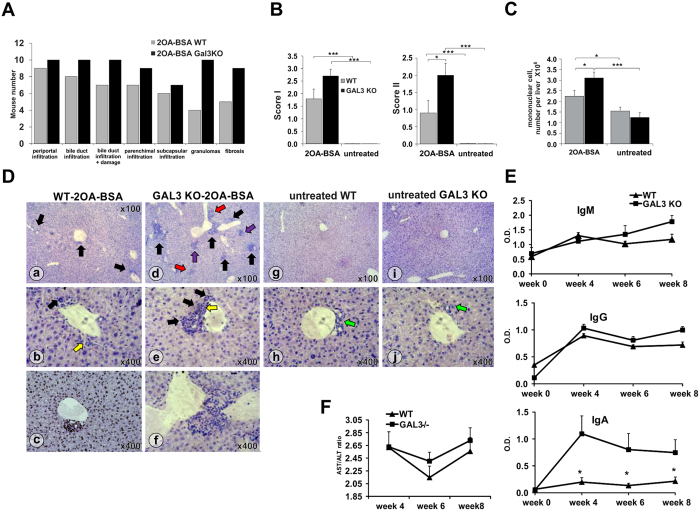
Deletion of Gal-3 enhanced PBC in C57BL/6 mice. (A) Number of mice with parameters of liver inflammation, granuloma formation, and fibrosis. (**B**) Total score I (liver infiltration and bile duct damage) and score II (liver fibrosis and granuloma formation). (**C**) Total number of mononuclear cells. (**D**) Representative liver sections of immunized WT (a–c) and Gal-3 KO (d–f); non-immuniezed WT (g,h) and Gal-3 KO (i,j) mice, arrows indicate: periductal mononuclear cell infiltrate (black); parenchymal infiltration (red); granuloma formation (violet); bile duct damage (yellow); bile duct obliteration (blue); no infiltration in control mice (green). (**E**) Serum levels of anti-PDC-E2 antibodies in non-immunized mice (week 0) and 4, 6, and 8 weeks after immunization with xenobiotic. (**F**) AST/ALT ratio in serum. Data presented as mean + SE, 9 mice per group, *p < 0.05, **p < 0.005.

To examine the role of Gal-3 in a mouse model of PBC, we immunized C57BL/6 WT and Gal-3 KO mice with the xenobiotic, 2OA-BSA, and measured the parameters of PBC. Untreated Gal-3 KO mice at 4 months of age did not develop any liver pathology (Fig. 2D). At eight weeks after immunization. 10/10 tested Gal-3 KO mice developed granuloma and 9/10 tested Gal-3 KO mice developed liver fibrosis. In contrast, only 5/10 of WT mice developed granuloma and 4/10 WT mice had fibrosis with 2OA-BSA treatment (Fig. 2A). This is similar to previous data on C57BL/6 mice^39^. Periportal infiltration was more pronounced in Gal-3 KO mice (Fig. 2A,D). There is no statistical difference in the degree of periportal inflammation, infiltration of bile ducts without damage, infiltration and damage of bile ducts, and subcapsular infiltrates (score 1), between Gal-3 K/O and WT mice (Fig. 2B). However, the extent of granuloma formation and fibrosis (score 2) was significantly higher in Gal-3 KO mice than WT mice (p < 0.05; Fig. 2B). Furthermore, the number of mononuclear cells was significantly higher in livers of Gal-3 KO mice (Fig. 2C) at eight weeks after disease induction (p < 0.005). Only small periductal infiltrates were observed in immunized WT mice (Fig. 2Da–c). In contrast, large inflammatory foci were present both around the bile duct and liver parenchyma in immunized Gal-3 KO mice (Fig. 2Dd–f). Obliterated ducts were frequently found in Gal-3 KO mice (Fig. 2Dc,f), but not in WT mice (not shown). We also found significant increase in serological anti-PDC-E2 IgG, IgM and IgA at four weeks after immunization in both groups of mice, with no difference in IgM and IgG levels between WT and Gal-3 KO mice (Fig. 2E). However, serum anti- PDC-E2 IgA antibody was significantly higher in Gal-3 KO mice compared to WT mice (p < 0.05) (Fig. 2E). There was no significant difference between AST levels; however, AST/ALT ratio in the sera was higher in the group of Gal-3 KO mice than in WT mice at weeks 6 and 8 after 2OA-BSA immunization (Fig. 2F), indicating more extensive liver damage in the absence of Gal-3.

### Influx of inflammatory CD8+ lymphoid cells is higher in the livers of Gal-3 KO mice

**Figure 3:**
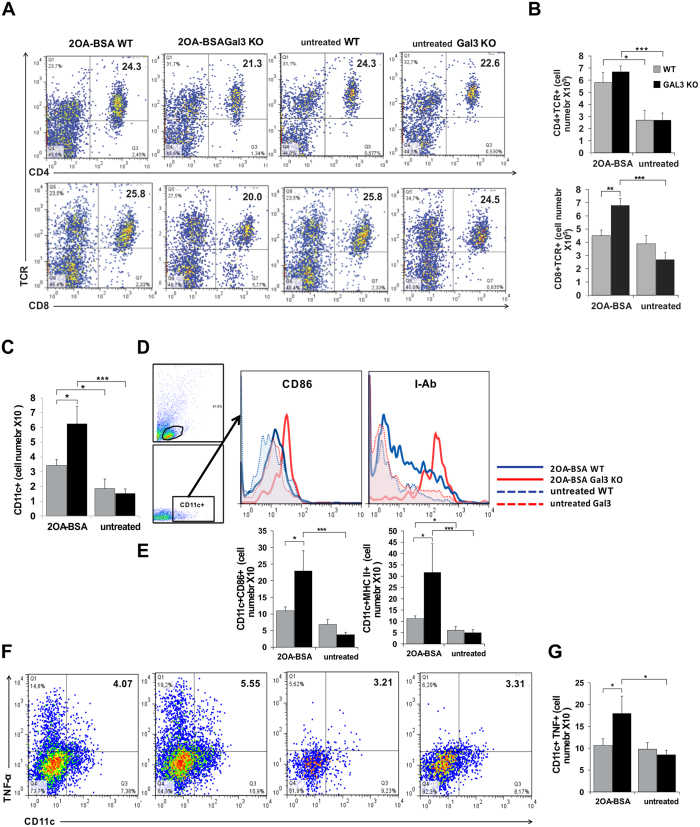
Gal-3 deletion leads to increase of CD8+ T lymphocytes and inflammatory phenotype of DCs in liver infiltrates after PBC induction. (**A**) Representative dot plots displaying the frequency and (**B**) total numbers of CD4+ and CD8+ T lymphocytes calculated per liver. (**C**) Total numbers of CD11c+ DCs in the livers. (**D**) Expression of markers of activation on CD11c+ gated cells. (**E**) Total numbers of DCs expressing CD86 and MHC II. Representative dot plots (**F,G**) total numbers of DCs expressing TNF-alpha. Data are presented as the mean+SE, 9 mice per group, **p < 0.005; *p < 0.05.

Eight weeks after 2OA-BSA immunization, there was no difference in the percentages of CD8+TCR+ and CD4+TCR+ cells in livers of WT and Gal-3 KO immunized mice, or between control and diseased mice (Fig. 3A). Although the total number of CD4+ and CD8+ T lymphocytes increased, only the number of CD8+ T lymphocytes was significantly higher in the liver of Gal-3 KO mice compared to WT mice (p < 0.005; Fig. 3B).

### Gal-3 deletion favors the proinflammatory phenotype of liver DCs

We examined the relative percentage of CD11c+ DCs in the livers of Gal-3 KO, WT immunized, and control mice. There was no difference in the percentage of CD11c+ DCs DCs between Gal-3 KO and WT mice, but significantly higher absolute numbers of DCs were detected in the livers of Gal-3 KO mice (Fig. 3C). DCs of Gal-3 KO mice had higher expression of markers of activation CD86 and MHC II molecules (Fig. 3D). Furthermore, a significantly higher number of CD11c+ cells expressing CD86 (p < 0.05) and MHC II (p < 0.05) molecules was observed in the livers of xenobiotic immunized Gal-3 KO mice (Fig. 3E). Percentage of CD11c+TNFα+ cells was higher in Gal-3 KO mice (Fig. 3G), but without significant difference. However, the absolute number of CD11c+TNFα+ cells was significantly higher in the group of Gal-3KO mice compared with WT mice (Fig. 3F; p < 0.05), confirming the inflammatory phenotype of DCs.

### Enhanced PBC in Gal-3 KO mice is characterized by more pronounced apoptosis of BECs

**Figure 4:**
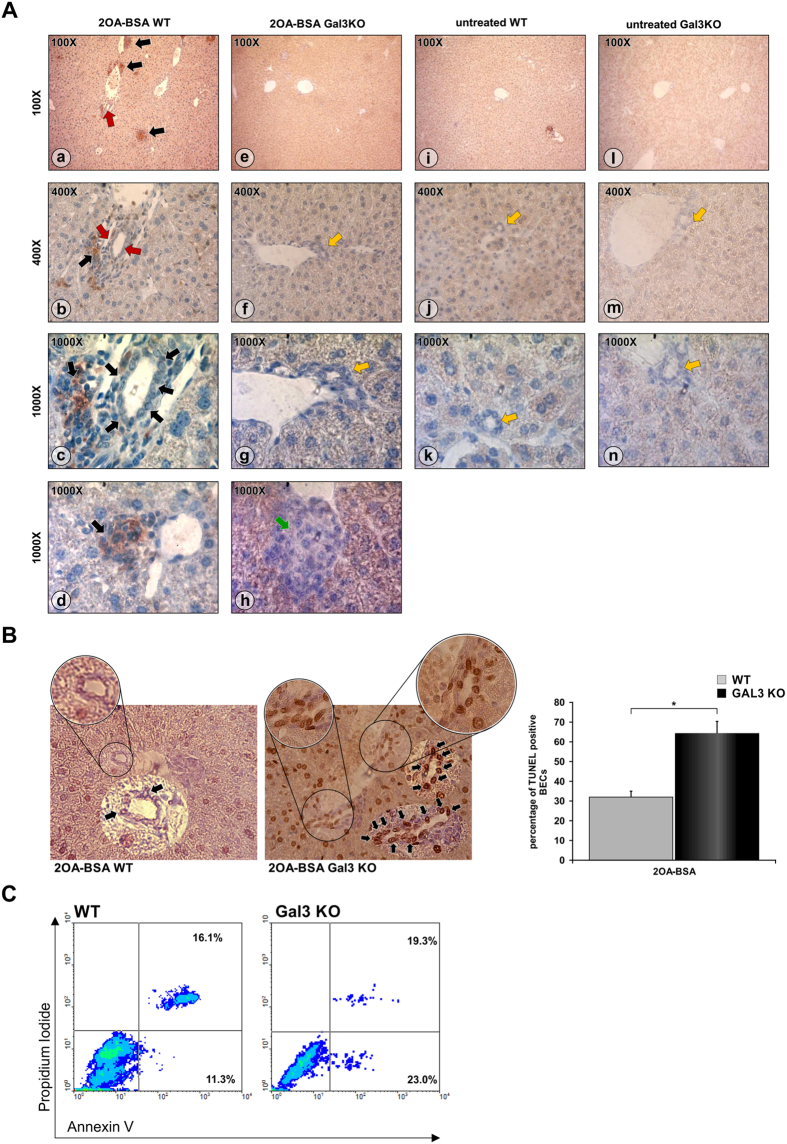
BECs of Gal-3KO mice are more susceptible to apoptosis. (**A**) Representative sections of Gal-3 liver immunohistochemistry. Gal-3 was expressed in BECs and mononuclear infiltrations in 2OA-BSA treated WT mice (a–c; red and black arrows, respectively) and granuloma (d; green arrow), but was not seen in the BECs, infiltrations (e–g; yellow arrows) and granuloma (h, green arrow) of 2OA-BSA Gal-3 KO mice and untretaed WT (i–k) and Gal-3 KO mice (l–n). (**B**) TUNEL staining of liver sections (arrows indicate apoptotic cells) and numbers of аpoptotic BECs presented as mean + SE; 9 animals per group; *p < 0.05. (**C**) Representative dot plots of apoptotic BECs isolated from healthy WT and Gal-3 KO mice, stained with Annexin V and propidium iodide after exposure to apoptosis inducer (ionomycin).

Consistent with previous reports and our data in humans we found that pathological changes in the liver were accompanied by increased Gal-3 expression in BECs. Immunostaining of liver sections showed no Gal-3 expression in untreated control mice (Fig. 4Ai–k), untreated mice, while significant increase in Gal-3 expression was detected in BECs and liver infiltrates in 2OA-BSA immunized WT mice (Fig. 4Aa–d). There was no Gal-3 expression in the livers of both untreated (Fig. 4Al–n) and immunized (Fig. 4Ae–h) Gal-3 KO mice. Next we analyzed the frequency of apoptotic BECs eight weeks after 2OA-BSA immunization. There were significantly more TUNEL positive BECs in the liver sections of Gal-3 KO mice compared with those in WT mice (Fig. 4B). To confirm the increased apoptosis of BECs of Gal-3 KO mice we isolated BECs from untreated WT and Gal-3KO mice, exposed them *in vitro* to apoptotic stimuli, and measured the percentage of apoptotic cells by flow cytometry. After 22 hours of *in vitro* exposure to ionomycin, we detected higher percentage of apoptotic (Annexin V positive) BECs isolated from Gal-3 KO mice, compared with those from WT mice (Fig. 4C).

### Enhanced bile duct damage is associated with enhanced Th1 immune response in the liver and enhanced systemic Th17 immune response leading to liver fibrosis

**Figure 5:**
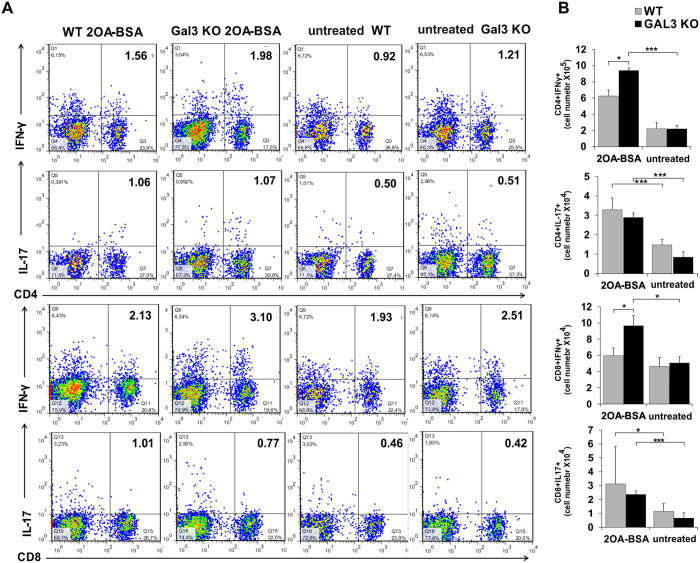
IFN-γ containing cells predominates in the liver of Gal-3 KO mice after PBC induction. (**A**) Representative dot plots of CD4+ and CD8+ cells positive for IFN-γ and IL-17. (**B**) The total cell numbers of CD4+ and CD8+ cells containing IL-17 and IFN-γ in liver in wild type and Gal-3 KO mice presented as the mean + SE; 9 mice per group, *p < 0.05, **p < 0.005.

Intracellular staining of liver mononuclear cells showed similar percentages of CD4+ and CD8+ cells containing Th1 and Th17 cytokines (Fig. 5A), but the absolute numbers of CD8+ and CD4+ cells containing IFN-γ was significantly higher (p < 0.05) in Gal-3 KO mice compared to WT mice (Fig. 5B). There was no significant difference in the total number of CD4+ and CD8+ cells containing IL-17 in the livers of Gal-3 KO and WT mice.

**Figure 6:**
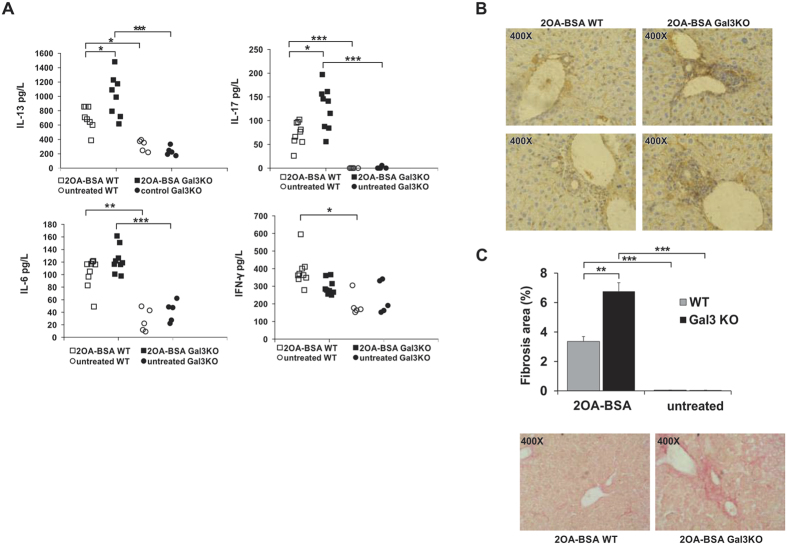
Gal-3 deletion increases serum levels of profibrotic IL-13 and IL-17 cytokines and fibrosis in 2OA-BSA induced PBC. (**A**) Serum levels of IL-13, IL-6, IL-17 and IFN-γ and (**B**) liver immunohistochemistry of CK-7 in WT and Gal-3 KO mice eight weeks after immunization with 2OA-BSA. (**C**) Liver fibrosis score of WT and Gal-3 KO mice and representative liver sections stained with Sirius red indicating minimal fibrosis in WT mice (black arrows) and marked fibrosis in Gal-3 KO mice (red arrows). Data are presented as the mean + SE; 9 mice per group, *p < 0.05.

Immunization with 2OA-BSA induces significant increase of serum levels of IL-6, IL-17, IL-13 in WT and Gal-3 KO (Fig. 6). Interestingly, the serum level of IFN-γ was statistically significantly higher in immunized compared to untreated WT mice, but not in between the immunized and untreated Gal-3 KO mice. Furthermore, there was no difference in the serum level of IFN-γ and IL-6 between xenobiotic immunized Gal-3 KO and WT mice, although Gal-3 KO mice had significantly higher (p < 0.05) serum levels of profibrotic cytokines IL-13 and IL-17 (Fig. 5C). Figure 6B,C illustrate the significant enhancement of bile duct damage and fibrosis upon xenobiotic immunization, as evaluated by anti-CK7 immunostaining and Sirius red staining, respectively.

## Discussion

Our previous studies indicated that deletion of Gal-3 attenuates several T cell mediated autoimmune diseases such as diabetes^4^, experimental autoimmune encephalomyelitis^3^, and inflammatory diseases including liver damage induced by T and NKT cells^7^,^8^. In contrast to these studies, we demonstrated that deletion of Gal-3 aggravated another autoimmune disease, PBC. The diverse effect of Gal-3 effect may be the result of the regulation of predominant mechanisms under different immunopathological conditions. Gal-3 can modulate the development of immunopathology on at least two levels: first, by modulating function of immune cells and second, by controlling various cellular functions. In this study, it is possible that Gal-3 deficiency is accompanied by enhanced xenobiotic induced apoptosis of BECs and subsequent enhanced release of putative antigen(s). In humans, expression of Gal-3 is negligible in normal liver hepatocytes and biliary epithelial cells^10^. This expression is significantly enhanced in pathological conditions such as cholangitis, cholangiocarcinoma^10^ and hepatitis B and C (Fig. 1).

In this study we provided experimental evidence that Gal-3 gene deletion leads to accelerated 2OA-BSA induced PBC. In C57BL/6 mice, Gal-3 deletion exacerbates the disease through significantly higher liver infiltrates of CD8+ T lymphocytes, with enhanced bile duct damage, liver fibrosis, serological level of PDC-E2 specific IgA and increased AST/ALT ratio (Figs 2 and 3).

Bile duct damage in humans with PBC directly correlates with the frequency of CD8+ T cells in the liver^40^. Predominance of CD8+ T cells in the portal tracts was also present in the IL-2Rα^−/−^ mice and 2OA-BSA induced mouse model of PBC^14^,^41^. PDC-E2 specific CTLs have the leading role in PBC pathogenesis as they are involved in the lysis of BECs^13^. More importantly, adoptive transfer of CD8+ T lymphocytes from dnTGFβRII mice induces autoimmune cholangitis with prominent inflammatory cell infiltration, bile duct destruction, and granuloma formation in the portal tract in Rag-1^−/−^ recipient mice, whereas transfer of CD4+ T cell did not induce PBC-like hepatic lesions^42^. Therefore, it is evident that an enhanced number of CD8+ T lymphocytes and more severe liver disease were developed in Gal-3 KO mice. In line with previous findings (39,40), higher AST/ALT ratio and more pronounced liver damage are observed in immunized Gal-3 KO compared with immunized WT mice.

The levels of anti-PDC-E2 in 2OA-BSA immunized WT mice are similar to those in the literature^39^. We also found increased serological anti-PDC-E2 IgG, IgM and IgA in the sera after immunization (Fig. 2E) without statistical difference in total IgM and IgG levels between WT and Gal-3 KO mice. However, there is a significantly higher level of anti-PDC-E2 IgA antibody in the serum of immunized Gal-3 KO mice compared to immunized WT mice (Fig. 2E). Rigopulou *et al*. reported that the hallmark of the disease is the presence of anti-PDC-E2 IgG, (41). However in Gal-3 KO mice, serum level of anti-PDC-E2 IgA appears to correlate with enhanced epithelial damage (Fig. 2E). This observation is of significance in light of the previous report on transcytosis of PDC-E2 IgA across the BEC and that such transcytosis initiated capase activated apoptosis of BEC^43^. The level of caspase activation directly correlates with anti-PDC-E2 IgA antibody in sera of PBC patients^43^. Taking into account this correlation, it is also noted that the elevation of anti-PDC-E2 IgA antibody level in the sera of 2OA-BSA immunized Gal-3 KO mice (Fig. 2) is in association with enhanced apoptosis of BECs of immunized Gal-3 KO mice (Fig. 4).

Gal-3 is expressed widely in epithelial and immune cells, such as gastric mucosa, colon mucosa, mammary epithelium, prostate epithelium, monocytes, and macrophages^44^,^45^. Expression of Gal-3 is often increased in epithelial and immune cells under inflammatory conditions. In this study, we showed that Gal-3 is expressed in BECs of patients with PBC (Fig. 1). In addition, we showed Gal-3 expression is absent in BECs of healthy mice but significantly increased in BECs of 2OA-BSA immunized mice (Fig. 4), indicating the relationship between Gal-3 expression and liver inflammation in 2OA-BSA immunized mice. Knockdown of Gal-3 sensitizes human colorectal cancer cells, keratinocytes, leukemia cells, human renal cell carcinoma and cholangyocarcinoma cells to apoptosis^46^,^47^,^48^,^49^ whereas overexpression of Gal-3 protects the cells from apotosis^50^. Hence, the expression of Gal-3 in the inflammatory liver of 2OA-BSA immunized mice may be a protective mechanism in the apoptosis of BECs.

It can be hypothesized that increased expression of Gal-3 in BECs in PBC is a compensatory mechanism to protect BECs from apoptosis induced by different stimuli in this disease. This hypothesis supports the finding of low expression of Gal-3 in BECs in later stages of PBC (data not shown) with marked fibrosis and progressive ductular damage.

Related to our finding presented in Fig. 5B, it appears that increased apoptosis of BECs in Gal-3 KO mice may facilitate release of autoantigens and induce stronger activation of DCs with higher influx of inflammatory lymphocytes, leading to enhanced bile duct damage (Fig. 6B) and liver fibrosis (Fig. 6C). Thus, enhanced apoptosis of BECs in immunized Gal-3 KO mice may be the consequence of the absence of Gal-3. BECs isolated from healthy Gal-3 KO mice which were exposed to apoptotic stimulus *in vitro* had significantly higher percentage of apoptotic cells compared with those from WT mice (Fig. 5B), confirming the protective role of Gal-3 against apoptosis of BECs.

Apoptosis of BECs is an important phenomenon in PBC^37^,^38^,^51^. When BECs undergo apoptosis, PDC-E2 remains immunologically intact and its expression at the apical surface of the small bile duct cells lining the bile duct lumen is confirmed by biliary immunostaining with selected PDC-E2-specific monoclonal antibodies^36^,^37^,^52^,^53^. PDC-E2 in its intact form is also localized in the apoptotic bodies of BECs where it is accessible to AMA recognition^37^. Thus it can be assumed that enhanced apoptosis of BECs from Gal-3 KO mice leads to enhanced exposure of autoantigen, consecutive enhanced stimulation of immune response and enhanced disease.

The other contribution of enhanced apoptosis of BECs in Gal-3 KO mice to more severe PBC could be through enhanced stimulation of innate immunity. Monocyte-derived macrophages from PBC patients incubated with apoptotic blebs from BECs in the presence of AMAs intensively produced proinflammatory cytokines^38^. In this study, proinflammatory CD11c+ DCs were present in the livers of Gal-3 KO mice (Fig. 6). This may be the consequence of stronger stimulation with apoptotic bodies in the presence of AMAs, even in the absence of Gal-3 in DCs.

An imbalance between the death of BECs and the proliferation of residual cells determines the progression toward bile duct loss and fibrosis. In advanced PBC, cholangiocytes lose their ability to proliferate and cell death by apoptosis prevails over compensatory proliferation. Apoptosis promotes fibrogenesis through activation of hepatic stellate cells (HSCs)^54^,^55^. We found enhanced fibrosis in livers of Gal-3 KO mice (Fig. 6). Gal-3 activates various profibrotic factors including fibroblast proliferation and transformation, and collagen production in various diseases^56^. However, our apparently conflicting result can be explained by significantly increased death of BECs in Gal-3 KO mice followed by enhanced activation of HSCs and enhanced fibrosis. Enhanced fibrosis in Gal-3 KO mice correlates with increased systemic levels of profibrotic cytokine IL-13 and IL-17 (Fig. 6)^57^,^58^. The increased number of CD4+ and CD8+ cells containing IFN-γ in the livers of Gal-3 KO mice (Fig. 5) is in accordance with a previous report that IFN-γ deficient mice immunized with 2OA-BSA had no inflammatory cell infiltrates in livers^59^. Higher numbers of IFN-γ containing CD4+ and CD8+ cells in Gal-3 KO mice is in line with enhanced inflammatory phenotype of DCs in Gal-3 KO mice. Enhanced liver inflammation, fibrosis and bile duct damage in Gal-3 KO mice immunized with 2OA-BSA suggest a protective role for Gal-3 in PBC, which can be of therapeutic relevance. Future advances in immunotherapy in PBC will require the consideration of many factors, including its multiple hit etiology and its evolving immune response at various stages of the disease^60^,^61^,^62^,^63^. Gal-3 is a particularly interesting target because of its promiscuous role in multiple inflammatory pathways.

## Methods

### Animals

Female Gal-3–deficient mice on the C57BL/6 background and wild-type (WT) C57BL/6 mice (8 weeks of age) were used for the experiments. Knockout mice were obtained from the University of California Davis (Davis, CA; by courtesy of D.K. Hsu and F.T. Liu). All of the animal procedures were approved by the Ethics Committee of Faculty of Medical Sciences, University of Kragujevac and conducted in accordance with the National Institutes of Health (NIH) guidelines for humane treatment of laboratory animals.

### PBC induction and evaluation of disease

Primary biliary cholangitis was induced as previously described^39^. Blood samples were colleted from the facial vein at weeks 2, 4 and 8 after initial immunization with 2OA-BSA, and tested for levels of anti-PDC-E2 antibodies using an enzyme-linked immunosorbent assay (ELISA) as previously described^39^. Briefly, 96-well ELISA plates were coated with 10 μg/ml of purified recombinant PDC-E2 in 100 μl of carbonate buffer (pH 9.6) at 4 °C overnight, washed with Tris-Buffered Saline Tween-20 (TBS-T), and blocked with 5% skim milk in TBS for 30 minutes. One hundred microliters of diluted sera (1:250) were added to each well and incubated for 2 hours at room temperature. After washing, horseradish peroxidase (HRP)-conjugated anti-mouse immunoglobulin (A + M + G) (H + L) (1:3,000) (INVITROGEN ZyMax™) was added. The plates were incubated for 1 hour at room temperature, the plates were re-washed and color developed with 100 μl of TMB peroxidase substrate (BD Biosciences, San Jose, CA) added to each well. Optical density (OD) was read at 450 nm at Zenyth multimode detector 3100. Previously calibrated positive and negative standards were included with each assay. Liver tissues were fixed in 4% paraformaldehyde at room temperature for 2 days and embedded in paraffin. Paraffin blocks were brought to room temperature and sectioned on a rotary microtome (Leica RM2135). Twenty four serial 4-pm sections were floated onto water at 40 °C before being transferred to glass microscope slides. The sections were deparaffinized, stained with hematoxylin and eosin (H&E) and every fourth (six slides) was evaluated for periportal inflammation, infiltration of bile ducts without damage, infiltration and damage of bile ducts, and subcapsular infiltrates. Based on the levels of pathology, the indices were scored as 0, none; 1, mild; 2, moderate; 3, severe; and 4, very severe pathology. Histological score I was calculated as mean value of each scored index. Granulomas, and fibrosis were scored as 0, none; 1, mild; 2, moderate; and 3, severe pathology and histological score II was calculated based on these values. Histological analysis and scoring were performed in blinded fashion. The images were captured with a light microscope (Olympus) equipped with a digital camera.

Sirius Red staining was done by treated rehydrated sections stained 1 hour with saturated picric acid containing 0.1% Sirius Red F3BA (Sigma-Aldrich, St. Louis). The slides were washed in two changes of acidified water, then dehydrated in three changes of 100% ethanol, and then washed in xylene. Quantification of fibrosis in mouse liver sections stained with Sirius Red (x10) was performed using ImageJ software (NIHh, Bethesda, MD), on 10 fields/section.

### Cytokine levels determination

Cytokine levels were determined using mouse duo sets for IL-17, IL-13, and IFN-γ (R&D Systems, Minneapolis, MN, USA) according to the manufacturer’s recommendations.

### Immunohistochemistry of mouse liver samples

Formalin-fixed, paraffin-embedded mouse liver tissue sections were incubated with rabbit anti-mouse cytokeratin-7 (CK-7) and Gal-3. Sections were visualized by rabbit-specific conjugate (Expose Rb-Specific HRP/DAB Detection IHC Kit; Abcam), and mouse-specific conjugate (Expose Ms-Specific HRP/DAB Detection IHC Kit; Abcam) and photomicrographed with a digital camera mounted on light microscope (Olympus BX51).

### Gal-3 detection in human serum and liver samples

The levels of Gal-3 in sera of PBC patients (11) and healthy controls (11) were determined using Human Gal-3 Quantikine ELISA Kit (R&D). Human liver biopsy samples were obtained from the Department of Pathology University of Kragujevac tissue collection and were stained with mouse antihuman Gal-3 antibody (Abcam, Cambridge, UK) and the mouse specific conjugate (Expose Ms-Specific HRP/DAB Detection IHC Kit; Abcam), according to manufacturer instructions.

### Isolation of hepatic mononuclear cells and flow cytometry

The isolation of liver-infiltrating mononuclear cells was conducted as previously described^8^. Mononuclear cells were stained with fluorochrome-conjugated antibodies, including CD4 (clone RM4-5), CD8α (clone 53-6.7), TCRβ (clone H57-597), F4/80 (clone T45-2342), CD11c (clone HL3), CD19 (clone 1D3), I-A/I-E (clone AF6-120.1), CD86 (clone GL1), IL-12 (clone C15.6), and TNFα (clone MP6-XT22) (BD Biosciences). Isotype Abs with matching conjugates were used as negative controls. For intracellular staining, cells were activated with PMA/ionomycin and processed as previously described. Live/dead cell discrimination was achieved by staining with Propidium Iodide, 10,000 events of viable cells were analyzed with the FACSCalibur Flow Cytometer (BD Biosciences), and analysis was conducted with FlowJo (Tree Star).

### Isolation of BECs and apoptosis assays

To isolate the intrahepatic BECs, a two-step liver perfusion was performed. The perfused liver was removed and hepatocytes were selectively removed by forcing them with gentle pressure through an incision in the liver. Cells were suspended in DMEM media supplemented with 10% FBS, 5% NuSerum IV (BD), 0.5 mg/ml insulin–transferrin–sodium selenite (Gibco), 1 mmol/l ascorbic acid 2-phosphate, 10K7 M dexamethasone (Sigma-Aldrich Corp.), 10 ng/ml EGF (R&D, Minneapolis, MN, USA), 10 ng/ml HGF (R&D, Minneapolis, MN, USA). After 10 days cells were exposed to ionomycine 1 μg/ml for 22 hours and percentage of apoptotic cells was determined by flow cytometry using the Annexin V FITC Detection Kit (BD Pharmingen, San Jose, CA).

### Statistical analysis

The data are presented as mean ± SD or mean ± SEM. Statistical significance was determined by Independent sample Student t test and ANOVA, and, where appropriate, Mann-Whitney U test or Kruskal-Wallis. Statistical significance was assumed at p < 0.05. Statistical analyses were performed using SPSS 13.0.

## Additional Information

**How to cite this article**: Arsenijevic, A. *et al*. Deletion of Galectin-3 Enhances Xenobiotic Induced Murine Primary Biliary Cholangitis by Facilitating Apoptosis of BECs and Release of Autoantigens. *Sci. Rep.* **6**, 23348; doi: 10.1038/srep23348 (2016).
